# Parents’ experience of child loss during pregnancy or birth: protocol for an explorative, sequential and participative mixed-methods study

**DOI:** 10.1186/s40900-025-00819-8

**Published:** 2025-12-05

**Authors:** Fritz Sterr, Julian Siepmann, Daniela Nuber-Fischer, Christian Rester, Karsten Gensheimer, Lydia Bauernfeind

**Affiliations:** 1 Deggendorf Institute of Technology, Faculty of Applied Healthcare Sciences, Land-Au 27, 94469 Deggendorf, Germany; 2 Faculty of Health, School of Nursing Sciences, Witten/Herdecke University, Witten, Germany; 3 Munich, Germany

**Keywords:** Fetal death, Experience, Miscarriage, Mixed-methods, Need, Parents, Pregnancy loss, Qualitative research, Spontaneous abortion, Stillbirth

## Abstract

**Background:**

Internationally, the rate of child loss during pregnancy or birth remains high. This has not only physical but also psychological implications for the parents. While much is known today about the medium- and long-term impact of such a loss, very little research is available on the experience and needs before and during intrauterine or perinatal loss. In addition, healthcare professionals feel insecure and unprepared when dealing with this specific group, which also has a lasting impact on those affected.

**Aim:**

To develop recommendations for healthcare professionals on how to deal with families before and during the intrauterine or perinatal loss of their child.

**Methods:**

An explorative, sequential and participatory mixed-methods study will be conducted. Along its design, an affected mother is involved throughout the entire study. In detail, workshops, focus groups and narrative interviews with parents are planned. We will include parents who have lost their child during pregnancy or birth within the last 12 months and live in Germany, Austria or Switzerland. The results of the research are then translated into recommendations, which are reviewed and confirmed together with affected parents in a Delphi survey. Finally, a guideline for healthcare professionals will be developed.

**Discussion:**

The co-creative design of this study enables the experience-based development of recommendations for healthcare professionals.

**Clinical trial registration:**

The study was registered in the National Library of Medicine on January 13^th^, 2025 with the unique ClinicalTrials.gov identifier NCT06771661 (https://clinicaltrials.gov/study/NCT06771661).

## Abstract (unstructured)

Losing a baby during pregnancy or at birth is still common worldwide, and it affects parents both physically and emotionally. While we know a lot about the long-term effects on parents, there is little research on what families go through *during* or *just before* the loss. Many nurses, physicians and midwives also do not feel confident or prepared to support families during these moments, which can make the situation even more difficult for those affected. This study aims to provide helpful guidance for healthcare professionals on supporting families during this challenging time. The researchers will talk directly with parents who recently experienced a loss in Germany, Austria or Switzerland. They will use interviews, group discussions, and workshops to understand what parents need most. In addition, a mother who has lost her child is involved in every part of the study. She advises and supports the researchers the whole time based on her own experiences. The findings will be turned into recommendations and reviewed with affected parents to make sure they are accurate and helpful. In the end, a guide will be developed for healthcare professionals to support families experiencing child loss during pregnancy or birth.

## Background

### Introduction

Worldwide, the number of stillbirths is estimated at about 2.6 million per year [1, 2]. While up to 26.4 stillbirths occur for every 1,000 live births in developing countries [3], in the USA, for example, the rate is significantly lower at 6 per 1,000 [4, 5]. Despite this difference, the rate of stillbirths remains high [4, 6] and still increases in some cases [7]. In addition, the risk of miscarriage also continues to stay on a high level, especially in the first trimester. Across all pregnancies, the rate of miscarriages is at 15% [8].

Today, many risk factors have been investigated that induce the pathological course of pregnancy. In addition to advanced age, ethnicity, maternal hypertension and malnutrition, the main factors for child loss during pregnancy are growth disorders, infections, cervical diseases, mental illnesses and substance abuse [5, 8–12]. Nevertheless, in over 60% of cases, the etiology of the child’s death remains unknown [13].

However, child loss during pregnancy not only has medical implications, but also a significant impact on the families affected. Research from the last 20 years has shown that a lack of support during pregnancy loss and insufficient efforts to deal with this traumatic event can have serious psychological and physical consequences for the parents [14–17]. In order to prevent these consequences, various assessments and interventions have already been empirically investigated [18–20].

### Problem definition

While much is known about the long-term effects of the loss of a child, a significant research gap is also evident at this point. Families express various needs during the intrauterine phase and their pregnancy loss (e.g. [21], ), but these are still not described and analyzed in a differentiated way. The experience before stillbirth (intrauterine phase) and during stillbirth (birth itself) is also not depicted in the literature, in contrast to the aftermath. This also means that there is a lack of explanations for the targeted development of interventions and the origin of long-term effects on the parents.

A closer look at international medical, midwifery and nursing literature also reveals that neither guidelines nor studies and textbooks contain recommendations on how to deal with parents during stillbirth. This is also evident in studies among healthcare professionals (HCPs). They often feel insecure when dealing with parents during stillbirth [22] and are confronted with the ambiguity of the situation in which they have to mediate between the parents and their needs on the one hand and the healthcare system, the facilities and their aims on the other [22]. Various challenges such as their own emotions and lack of knowledge influence the work of HCPs [23] and lead to parents being avoided and stigmatized [22].

HCPs state that they have little or no training in caring for this specific patient cohort [22]. They suffer during the care themselves [22] and experience stress, especially when they have little experience or knowledge and perceive the missed care or inadequate staff performance [24]. HCPs are longing for an in-depth understanding, multi-professional training and sensitization tailored specifically to this patient cohort [23].

This situation results in inadequate care for the parents. They feel poorly informed and have few opportunities to communicate with HCPs [25, 26]. Finding themselves in a highly challenging situation with special needs [27], parents often feel overlooked and experience indifferent or dismissive HCPs, which leads to anxiety and frustration [28]. These experiences along with the lack of interaction with HCPs shapes the parents not just temporarily, but for years [23, 26, 27].

Parents are unable to assess and properly understand their own situation and the little information available [28], which can result in their (gradual) social withdrawal, psychological problems and an unhealthy lifestyle as a coping strategy [28, 29]. In various countries, there is also a lack of follow-up care, guidance and bereavement support beyond the acute inpatient stay [26, 30].

### Aim and research question

Based on the problem definition, this study aims to develop recommendations for HCPs on how to deal specifically with families before and during the intrauterine or perinatal loss of their child. To this end, two research questions were formulated that are to be answered by the study:

- How do parents experience the intrauterine or perinatal loss of their child?
- What needs and requirements arise for families experiencing intrauterine or perinatal loss of their child?

## Methods

In order to answer the underlying research questions and achieve the formulated aim, an explorative-sequential mixed methods study will be conducted [31]. The study is designed as participatory research and, in the sense of a co-creative design, will not only survey affected parents in three central survey steps, but also actively involve them in the development of the study design and the progress of the study. To enhance transparency over the study course, we registered our study in the National Library of Medicine (https://clinicaltrials.gov/study/NCT06771661) with the clinical trial number NCT06771661.

The reporting of this study protocol follows the ‘Standard Protocol Items: Recommendations for Interventional Trials’ (SPIRIT) guideline [32, 33], as no other reporting guideline could be identified for study protocols. Nevertheless, the SPIRIT guideline is designed for interventional trials, which is why not all recommended items fit for our study and will therefore be excluded.

### Study setting

In order to de-pathologize stillbirth, none of the data collection will take place in the hospital setting. The workshops, focus groups and the ensuing Delphi method will be set up on the research institutions involved. The narrative interviews will be conducted at the parents’ homes or at a neutral location of their choice.

### Eligibility criteria

As recommended for primarily qualitative studies [34, 35], our eligibility criteria is based on the PICo design (**P**opulation, Phenomenon of **I**nterest, **Co**ntext). The population of interest includes parents (mothers and fathers) after stillbirth who are at least 18 years old, understand and speak German and live in Germany, Austria or Switzerland. They must also have legal capacity and participate in the study voluntarily.

The phenomenon of interest encompasses the experience of the parents as well as the needs and requirements of the family (mothers, fathers, siblings). In order to avoid possible recall bias [36, 37], the intrauterine or perinatal loss must not have occurred more than twelve months prior to data collection.

The context is stillbirth, which in this study is defined as the birth of an intrauterine deceased embryo or fetus, regardless of its age. In addition, we included parents after a (largely) physiological birth with perinatal death of the newborn child. In terms of the time period, we deliberately did not set any posterior limits as eligibility criteria but rather focused on a qualitative definition by the participants themselves. When recruiting participants, the phrase ‘death of the child during pregnancy or birth’ is therefore used. We deliberately did not distinguish between early or late loss during recruitment, as we want to uncover both potential differences and similarities in their experiences throughout the study. Parents relating to the phenomenon are eligible to participate in the study.

### Sample size

In accordance with the recommendations for qualitative interviews [38–43], we estimated 25–30 participants for the workshops and focus groups. For each workshop day and focus group, eight to ten participants should be recruited. For the narrative interviews, which focus on in-depth content rather than quantity, we plan to recruit eight to twelve participants. In order to subsequently enable a sufficient Delphi process in which every person has their say, we will recruit twelve to 16 participants [44–46].

### Recruitment

We will primarily recruit participants for our study using a digital snowball principle. Therefore, a landing page was set up via the primary affiliation of the authors, on which all relevant study-information can be found (https://www.th-deg.de/sterneneltern). The university’s marketing service creates advertising materials. These will be distributed to relevant healthcare institutions via email or direct message with a request for forwarding. The study will also be publicized on social media (Facebook, Instagram, Twitter) via the university’s institutional channel. In addition, the aforementioned organizations will be asked to advertise the study on their own social media channels. In case of only a few participants being recruited via this approach, information material will be sent to midwife practices, birth centers, gynecologists, obstetrics departments in hospitals and support groups.

If a potential participant contacts the research team, an initial preliminary telephone interview takes place in which the intention of the study is explained. In addition, background information is queried and it is checked whether the participants actually correspond to the required eligibility criteria.

### Methodological framework of the study

The entire study is planned as a participatory research project and draws on the central premises and recommendations of Hartung et al. [47]. The aim is to organize the entire research process in a participatory manner, from reflecting on the current situation to identifying the research gap, formulating the aims and questions, designing the study and its methods, collecting and analyzing the data and publishing the results.

To this end, one participatory researcher (DNF) has been an integral part of the research team since the start of the study in March 2024 and is involved in all process steps and decisions, including this study protocol. The participatory researcher has no scientific training and works as a consultant for parents after stillbirth, being a mother of a stillborn child herself. She is encouraged to contribute her own experience and expertise from her work to the entire research process.

In addition to the joint agreements within the entire project team, the researchers also hold internal meetings to prepare and follow up on the discussions with the participatory researcher. The overarching concept of the project thus follows the understanding of participatory research, i.e. conducting studies not on, but with the people whose lived experience and living conditions are being researched [48].

In order to further emphasize the participatory approach and enable the research subject to be linked back to the affected group, this study also uses an experience-based co-design [38]. The aim of this approach is to better understand the experiences of those affected in order to improve healthcare and cooperation between HCPs. The focus is on those affected themselves and their families. In terms of implementation, this co-design is divided into two central phases: the 1) discovery phase and the 2) co-design phase.

**Fig. 1:**
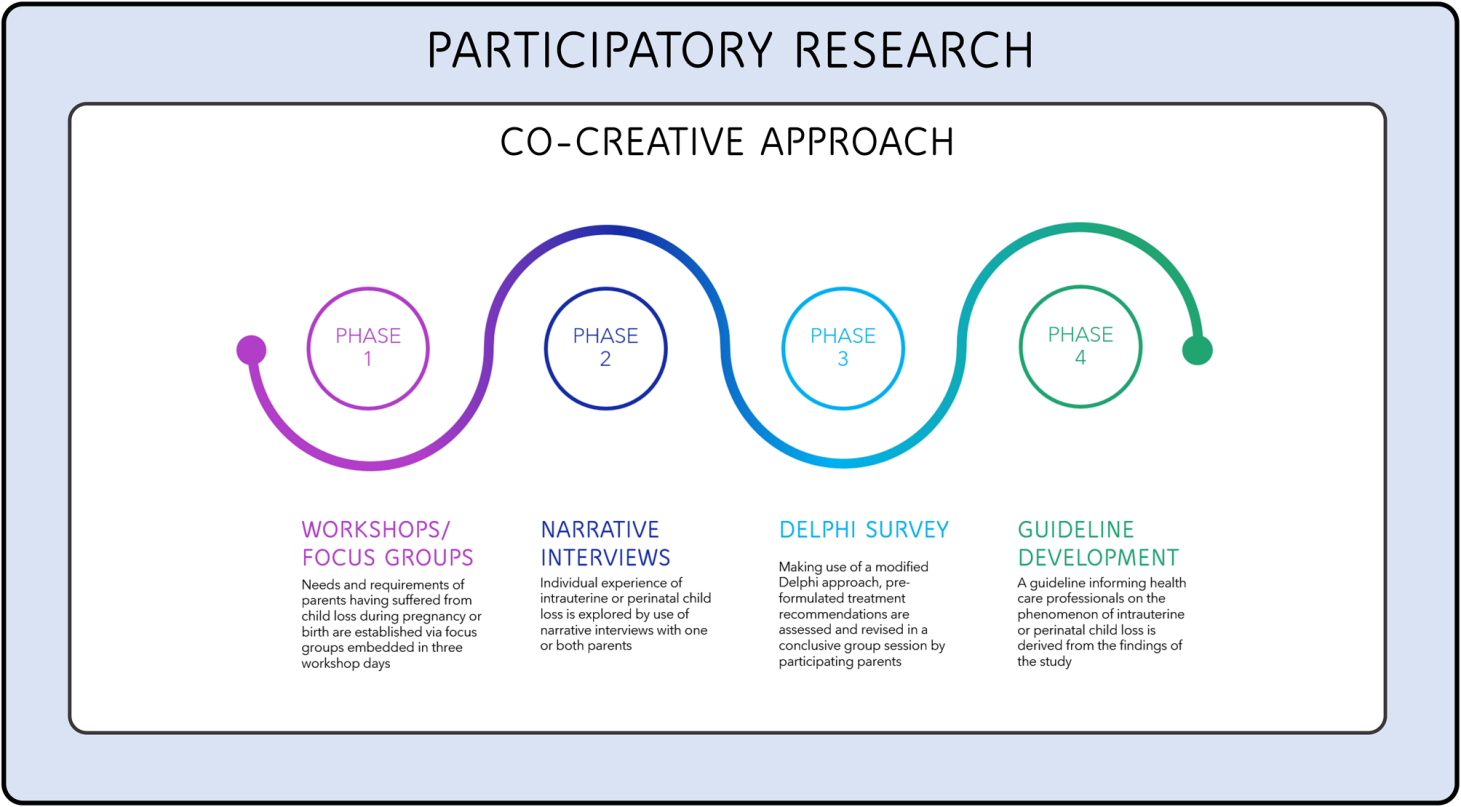
Methodology of the study on parents after child loss

In our study, after the general conception of the study with a participatory researcher, a comprehensive workshop with affected parents is planned (phase 1), in which the breadth of the topic and central aspects will be defined. The focus group interviews that take place at the end of the workshop are intended to reveal the needs and requirements of those affected. The results and missing data will then be considered in the subsequent narrative interviews (phase 2), which focus on the lived experience of parents during their loss. Provided that a sufficient number of participants can be recruited, no individuals who have already participated in the focus group interviews will be selected for the individual narrative interviews to avoid potential biases. Finally, recommendations will be developed from the entire data analysis and are to be agreed by affected parents within a Delphi survey (phase 3). This process ensures a continuous user-based development of a guideline for health professionals (phase 4), considering the experience, needs and requirements of those affected. The overarching methodological framework for our study and its phases are depicted in Fig. 1.

### Data collection methods

Based on the participatory, co-creative premise of this study, a workshop [40] will take place at the beginning of the research process, which will be repeated on a total of three days with different samples in Bavaria, Germany. As this study receives no financial support, we are unable to reimburse transportation expenses. Nevertheless, food, drinks and additional catering for the whole day will be provided on site. Eight to ten people per day who have lost a child themselves as a mother or father along the formulated inclusion criteria will take part in this workshop. The workshop is structured in three parts: 1) opening, 2) world café, and 3) focus group interview. In order to create a pleasant and trusting atmosphere, the researchers first introduce the study and themselves and then explain the general conditions of the day (dealing with each other, no right/wrong, everything remains in a protected space).

The participatory researcher will also be present throughout the workshop day as an additional contact person and confidant for the participants. Afterwards, the participants can tell their own story, if they wish. Thoughts, words and sentences from the participants are then collected on a large pinboard, which they formulate themselves in response to the question ‘What comes to your mind when you think back to the loss of your child?’. This session serves as a warm-up and brainstorming session. Participants should familiarize themselves with the associations of others and thus be stimulated in their own thinking.

#### World café

The participants are then divided into two small groups. Following the World Café method [49], which focuses primarily on the discussion among participants and the resulting thoughts and ideas, three stations are designed, each of which is attended by a small group for 30 minutes and moderated by researchers. The first station comprises the hierarchization of various needs and requirements of people identified in studies (e.g. communication, self-efficacy, safety). Two thematic scales (relevance, actual consideration) are laid out in the room; there is only a minimum and a maximum, but no fixed scaling. The small group should intuitively allocate the needs on this scale and justify what is placed where and for what reason. The researchers moderate this station and take field notes.

At the second station, the parents deal with public awareness of the phenomenon. Three short extracts from newspapers are used to present public narratives about parents after stillbirth or loss during birth, which the participants are then asked to assess critically. This is followed by questions about their own perception and identity: How is the topic of stillbirth currently debated in public? How do you want the loss of a child during pregnancy or birth to be covered in the media? The researchers again take on a moderating role and prepare field notes and audio recordings.

The third station addresses the ideal-typical care of affected families before and during the loss experience. A creative method is used here. In the first 20 minutes, the participants are given the opportunity to create a picture using the materials provided or to develop a poem or text. In the last 10 minutes, the developed artworks are then presented to the group. The researchers take photos of the results and field notes during the conclusive plenary presentation.

#### Focus groups

At the end of the workshop days, focus group interviews will be conducted in line with the methodological recommendations of Krueger [39]. These will last 60 to 90 minutes and focus on the needs and requirements of parents before and during the intrauterine or perinatal loss of their child. The focus group interviews serve to answer the second research question and provide a basis for the subsequent derivation of treatment recommendations.

Specifically, a semi-structured guideline is used for the focus group, which contains central topics, but is designed to enable a dynamic course of the interview and the addition of further topics. Starting with the introductory question about the general needs during the loss while pregnancy or birth, various core aspects (flow of information, the birth moment itself, expectations, handling strategies) are then addressed and concluded with a question about the ideal type of care. The interviews will be analyzed using Mayring’s qualitative content analysis [50] in the MAXQDA software.

#### Narrative interviews

In order to answer the first research question concerning the experiences of affected parents, narrative individual and partner interviews will be conducted. Depending on the number of participants in the focus group, eight to twelve interviews are planned with individual or both parents. If possible, the interviews should take place at the participants’ homes or at a location chosen by the interviewees, as long as this provides a quiet and private atmosphere.

The narrative interviews follow the methodological recommendations of Schütze [42]. Since participants usually want to tell their story in its entirety, narrative interviews often take a long time. However, not every detail of the story is relevant to the research. Despite the open nature of a narrative interview, the interviewer must therefore establish a certain sequence, create links between events, and continuously weigh and evaluate individual statements and situations in relation to the overall message of the story without interrupting the flow of the interviewee´s speech [41, 42]. In five steps, starting with a brief introduction and reflection on the project (explanation phase), a narrative impulse is given (introduction phase): ‘Mrs/Mr X, you recently lost your child during pregnancy/during birth. Please tell us what it was like for you. What did you experience during the loss experience?’ The subsequent narrative phase forms the main part of the narrative interview and can last indefinitely. The interviewer acts primarily as a listener and repeatedly gives the interviewee signals of agreement and understanding in order to maintain a pleasant atmosphere. The interview then moves into the inquiring phase when the main narrative is finished. The interviewer then asks questions and asks the interviewee for clarification, details or categorization of the narrative. In the final summarizing phase, the interviewer primarily aims to provide an overarching summary of the experience and asks for an assessment: ‘What consequences did this event have for your future life?’ [40, 41, 43].

#### Delphi approach

Once the focus group interviews and narrative interviews have been analyzed, the research team will translate the findings into treatment recommendations. These are directed towards HCPs (physicians, nurses, therapists, midwives) who are involved in supporting and caring for parents before and during the intrauterine or perinatal loss of a child. The recommendations in the form of individual shorter or longer statements are to be agreed and further developed with affected parents in the sense of participatory, co-creative research. The Delphi method, which will be used as the final data collection instrument and represents the quantitative component of this mixed-methods study, is particularly suitable for this purpose.

Methodologically, a slightly modified form of the classic Delphi method is used [44–46]. Twelve to 16 affected parents are invited to the researchers’ university as experts to assess, evaluate and further develop the pre-formulated treatment recommendations in an iterative process over the course of a whole day. In preparation for this, the treatment recommendations will be sent to all participants two weeks in advance so that they can familiarize themselves with the formulations before the Delphi process. If necessary, several rounds will take place on the day itself. Each round consists of 1) appraisal, 2) discussion and 3) revision of the recommendations, and ends with a break.

In detail, the recommendations are to be evaluated anonymously via an online platform [51, 52]. This enables immediate visibility of the results and transparent mapping of the group’s assessment. After the assessment, the participants are free to briefly comment on the individual and controversial recommendations. The recommendations are then revised by the researchers on the basis of the discussion and put to the vote again. The aim is to achieve the greatest possible consensus or consensus over dissent [53], while adhering to the quality standards and criteria [54]. The Delphi rounds are repeated until this goal is achieved. If the time frame of a single session should prove to be insufficient, a follow-up date is to be agreed with the participants.

### Ethical approval

As this study is concerned with a vulnerable group, we had an intensive exchange with the Ethics Committee ‘Gemeinsame Ethikkomission der Hochschulen Bayern’ (Bavaria, Germany) in summer 2024 to conform ethical principles for social research. In particular, an emergency strategy was developed to provide study participants with psychological support and referrals to additional support services during and after the study if required. Throughout the study, a family counselor accompanies the parents and is available as a person of trust and contact person. If psychological emergencies or crises do occur, a nursing professor specialized in mental health as well as a professor and psychiatrist are on call for the patients at all times. In November 2024, we received a positive ethics vote for our study (Number: GEHBa-202409-V-237-R2).

### Data privacy management

Over the course of the study, we will collect several personal and health-related data from the participants. Specifically, we will ask about their age, gender and the country of residence. We also want to know when the loss of the child took place (month, year) and whether the child died during pregnancy or birth. Finally, we will ask whether the affected parents gave birth to other children before and after the stillbirth. We deliberately refrain from collecting further personal data (school-leaving qualifications, education, place of residence, financial situation, etc.). This data does not add any value and in no way contributes to answering our research questions.

Nevertheless, we will collect several sensitive data that are particularly in need of protection. To secure this data, we have gone through a data privacy management process with the data privacy officers of the authors’ primary affiliation and jointly developed a strategy. The final results are publicly available (https://dpm.th-deg.de/infoduties/KmsaRM).

Consequently, all participants must sign an informed consent before participating in the study. In addition, they can withdraw from their participation at any time and are able to request the deletion of all contributions. All data collected (on paper, as audio, as images) is pseudonymized and stored digitally in the primary affiliation’s double-protected cloud. A key list is created for decoding, which is locked away as a printed version. Only the authors of this study have access to the pseudonymized data. All of them have signed a confidentiality agreement and guarantee that no information will be shared with third parties.

### Dissemination policy

We plan to publish all steps of the data collection (workshops, focus groups, narrative interviews, Delphi) and make our findings available to the public as open access articles. We also want to present the results to the scientific community and HCPs at relevant scientific conferences and congresses. We are also planning to contribute to the guideline development in relevant medical societies. We will also contact healthcare facilities and present the recommendations to them. To this end, we will offer free training courses from the university. Finally, we are also open to other institutions, such as non-healthcare organizations and employers who have employed parents with such a loss.

## Discussion

We anticipate a high level of interest in our study overall, both from affected individuals and from HCPs. In preliminary discussions with hospital staff and other healthcare facilities, we learned that there is a great deal of uncertainty in many places about how to deal with the affected parents. The treatment recommendations will be able to provide an important answer to this and depicted situation in the background.

Parents having experienced stillbirth are also indicating via various media channels (social media, newspaper articles, interviews, podcasts) that there is a need for such a study that enables them to be seen and heard. With our participatory, co-creative approach, we make a significant contribution to ensuring that this group of people is actively involved in a research project and receives more attention in the scientific community.

## Data Availability

No datasets were generated or analysed during the current study.

## Abbreviations

GEHBa: Gemeinsame Ethikkomission der Hochschulen Bayern
HCP: Healthcare professionals
